# Case report: Mature extragonadal teratoma at the proximal part of the tail in a kitten

**DOI:** 10.3389/fvets.2022.1003673

**Published:** 2022-11-21

**Authors:** Sirintra Sirivisoot, Naklop Siripara, Nlin Arya, Somporn Techangamsuwan, Anudep Rungsipipat, Tanit Kasantikul

**Affiliations:** ^1^Center of Excellence for Companion Animal Cancer, Department of Pathology, Faculty of Veterinary Science, Chulalongkorn University, Bangkok, Thailand; ^2^Pet Pro Veterinary Clinic, Bangkok, Thailand; ^3^Department of Pre-Clinic and Applied Animal Science, Faculty of Veterinary Science, Mahidol University, Nakhon Pathom, Thailand; ^4^Clemson Veterinary Diagnostic Center, Clemson University, Columbia, SC, United States

**Keywords:** cat, extragonadal, histopathogy, mature form, teratoma

## Abstract

An 8-month-old, intact male, domestic shorthair cat was referred for a mass on the proximal ventral part of the tail which had been found since the animal was born, and due to the presence of a linear fissure with rows of ectopic teeth, the veterinarian suspected that the mass had recently ruptured. Tail amputation was elected and the entire mass was successfully surgically excised. From the gross examination, this mass had an open cyst-like structure with a prominent area composed of hair, teeth, and bone. Histopathology revealed two components of germinal layers including hair follicles, adnexal tissue, neural tissue, teeth, muscle, fat, bone, and lymphatic vessels. The histopathological diagnosis was consistent to mature teratoma. Although, complete excision could not be definitively confirmed histologically, this kitten is currently well and has not developed any recurrent mass at the surgical site after 2 years of post-operation.

## Introduction

Teratomas are germ cell tumors and are rarely reported in domestic animals. The unique feature of this tumor is the presence of multiple embryonic germ layers including ectoderm (hair, teeth, nervous tissue), mesoderm (fibrous or adipose tissue, bone, muscle, cartilage) and endoderm (respiratory tissue, salivary gland). Teratomas commonly occur in an ovary or cryptorchid testicle; however, a few cases of extragonadal teratomas have been reported in cats at retrobulbar, head, perineal and coccygeal areas (1–4). Based on the histomorphology, it can be subdivided into immature and mature variants, depending on the degrees of differentiation of neoplastic components toward normal tissue structures. Most mature tumors are benign, and surgical excision with a clear margin is usually curative (2), while neoplasms with immature components can be malignant with metastatic potential (5, 6). In the present case, we reported a case of a mature extragonadal teratoma in a male kitten with a clinical outcome after treatment along with a brief review.

## Case description

**Figure 1:**
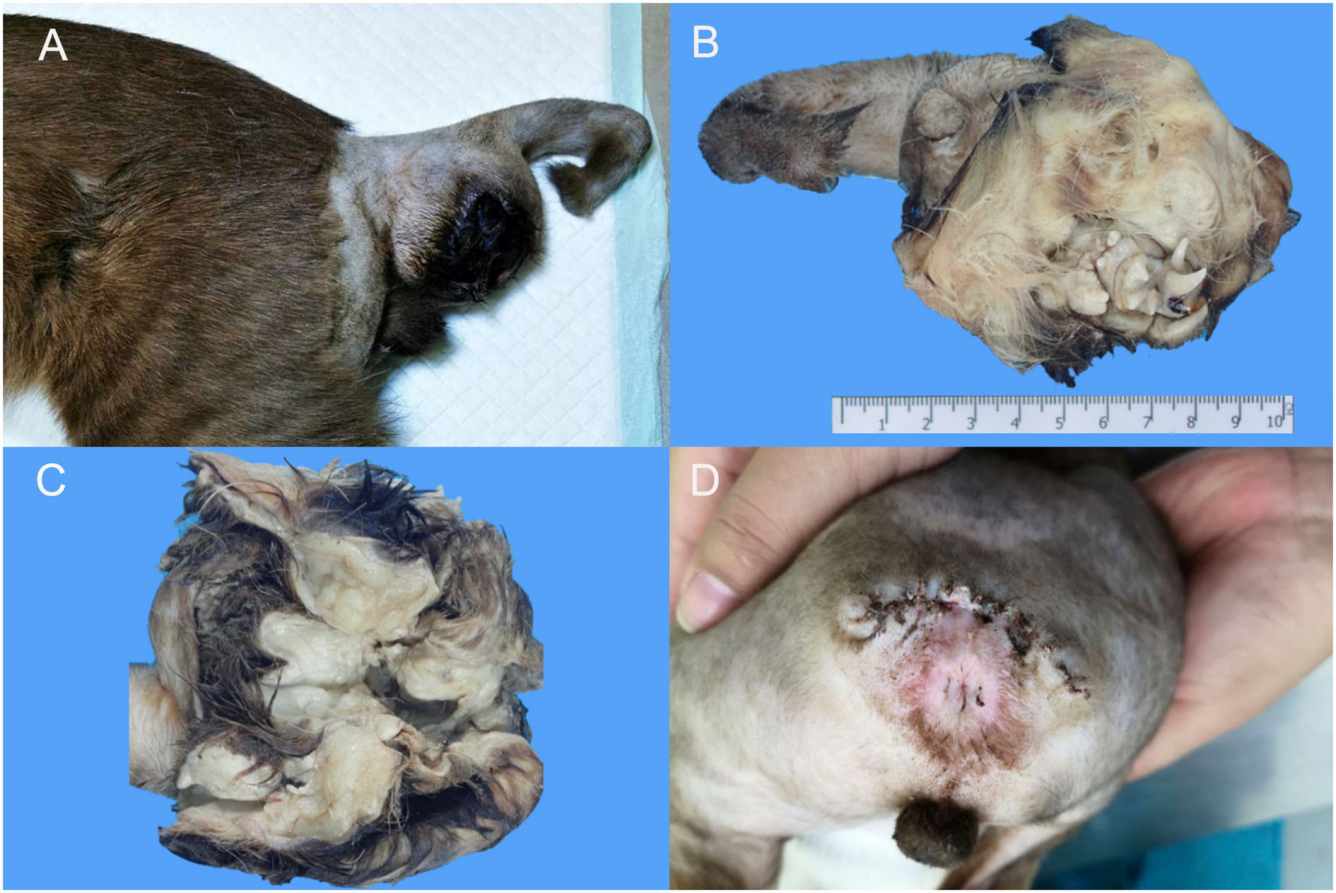
Macroscopic findings of extragonadal teratoma at 1st−8th caudal vertebrae in a male kitten. **(A)** A firm, well-circumscribed mass with size ~4.8 × 8.5 × 4.5 cm was located at the ventral part of the caudal vertebrae. **(B,C)** The cross section of the mass revealed hairs, teeth and multiple cystic structures. **(D)** The surgical wound after suture removal.

An 8-month-old, domestic short hair, intact male cat was presented to a private animal hospital with a chief complaint of a slow growing skin mass on the ventral tail between the 1st to 8th coccygeal vertebrae (Figure 1A) observed since the animal was born. The mass gradually grew and finally ruptured. Upon the physical examination, the kitten had normal vital signs with a flea infestation, and both testicles were in the scrotum. The size of skin mass was 4.8 × 8.5 × 4.5 cm and was firm and expansile, apparently not invading the surrounding tissue nor sacral or coccygeal bones. Based on the location and mass size, tail amputation was ultimately elected. The entire mass was successfully removed and was submitted for histopathological examination. The surgical wound healed, and the stitches were removed 10 days after the operation (Figure 1D).

The mass was submitted to the Veterinary Diagnostic Center, Faculty of Veterinary Science, Mahidol University for histopathologic examination. Upon gross dissection, the mass revealed a large central space with associated clusters of canine, premolar and molar teeth and small bones scattered over haired skin. The associated multilobulated bulks of adipose tissue, comprising the sac wall (Figures 1B,C), was located at the ventral part of the caudal vertebrae. The whole amputated tail along with the mass was fixed in 10% neutral formalin for at least 24 h. Several specimens through the soft tissue mass including the proximal margin of the amputated tail were trimmed, routinely processed for histology, embedded in paraffin blocks, cut into 4–6 μm-thick sections, and then stained with hematoxylin and eosin (H&E). Some sections through the sac wall with bony texture and tooth were cut for histopathologic examination after complete decalcification with Cal-Rite decalcifying solution (Thermo Scientific, MI, USA).

**Figure 2:**
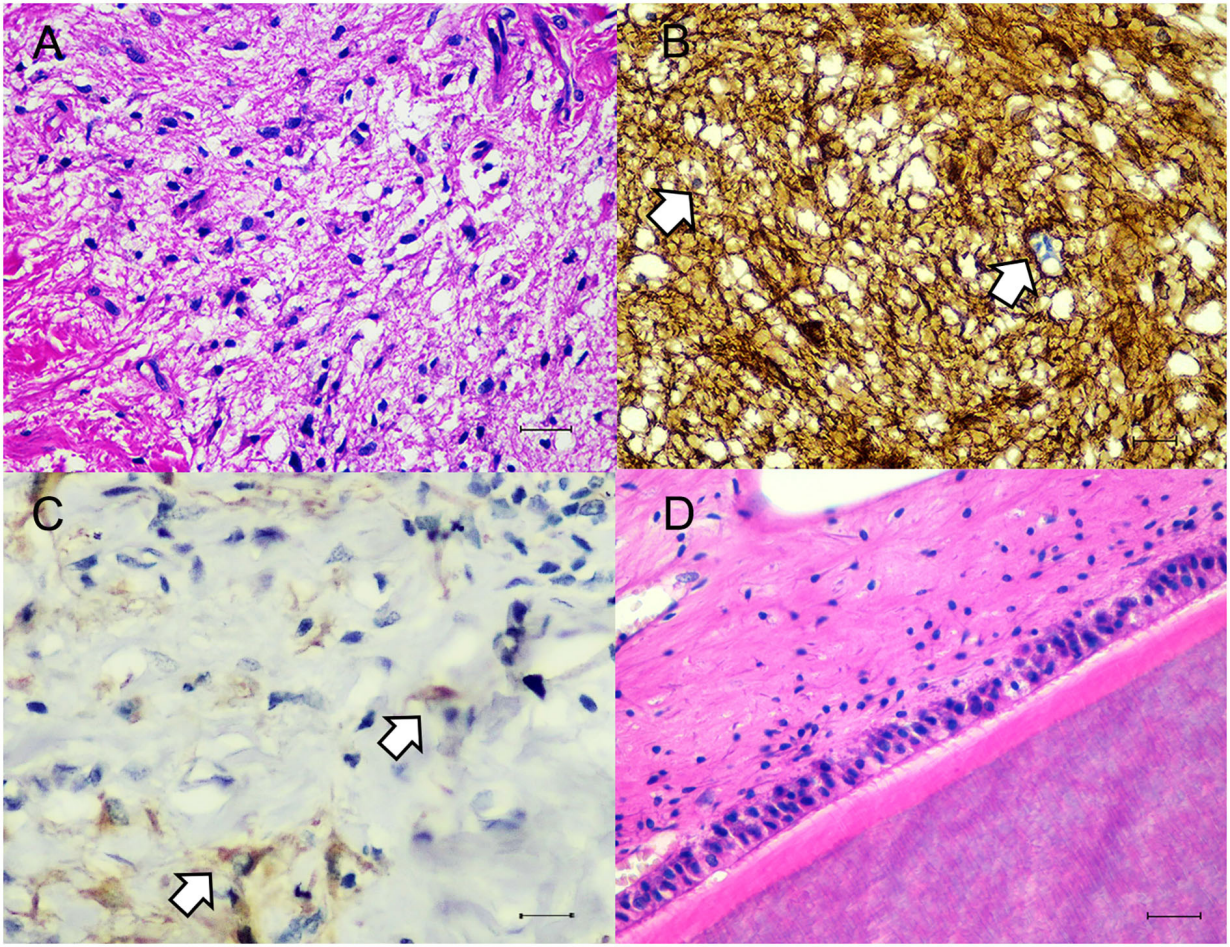
Microscopic findings of ectodermal components. **(A)** The neuronal tissue was composed of many glial cells amid fibrillar to neuropil. H&E. Bar = 50 μm. **(B)** Glial cells showed immunostaining with GFAP (white arrows). IHC. Bar = 50 μm. **(C)** Neurons showed immunostaining with Neuron-n (white arrow). IHC. Bar = 50 μm. **(D)** Area of tissue differentiation toward tooth characterized by large island of eosinophilic dentin and enamel with palisading layer of ameloblasts amid variably dense periodontal ligament stroma. H&E. Bar = 50 μm.

**Figure 3:**
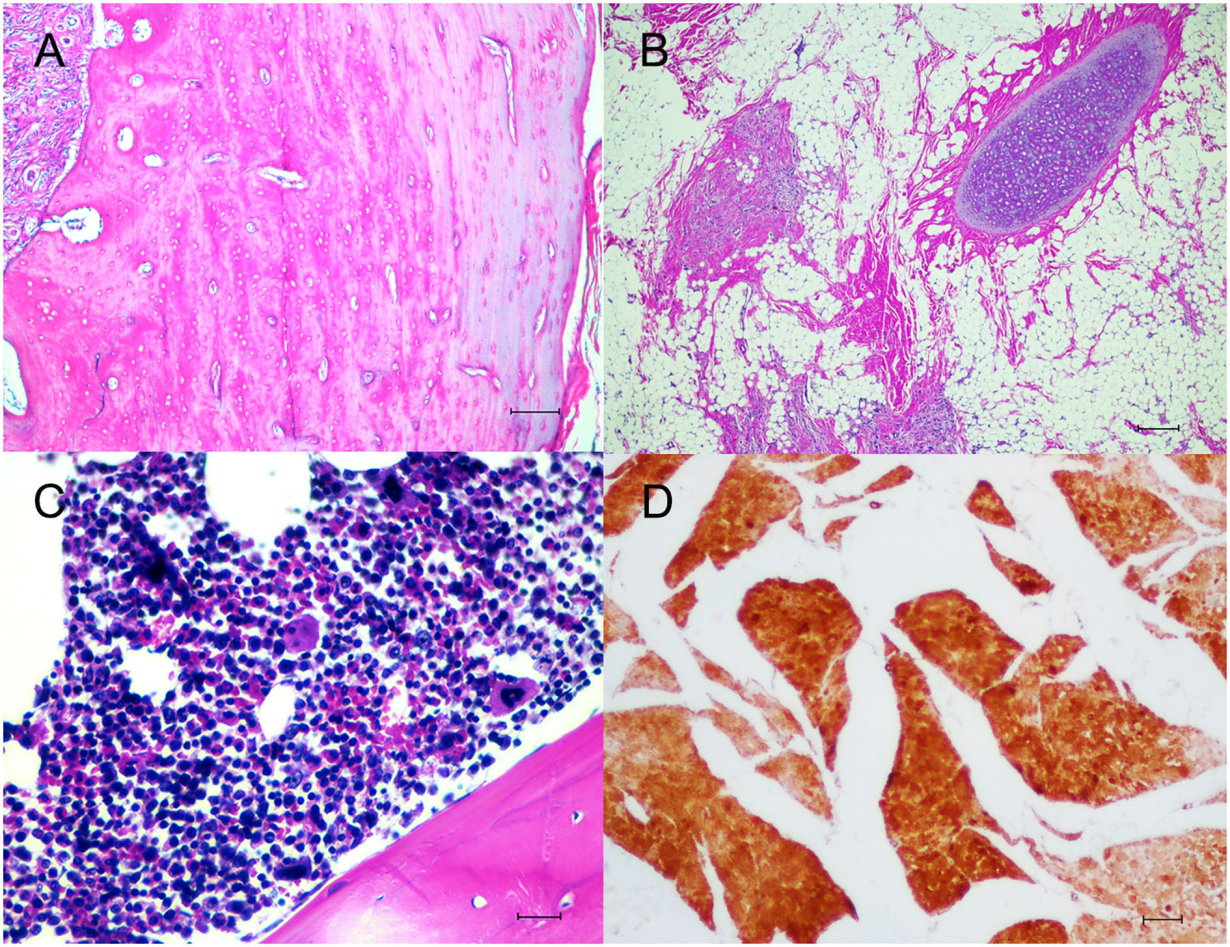
Microscopic findings of mesodermal components. **(A,B)** The mesodermal tissue consisted of cartilages, adipocytes, smooth muscles and bony trabeculae. H&E. Bar = 100 μm. **(C)** The proximal margin contained pools of hematopoietic cells. H&E. Bar = 50 μm. **(D)** Smooth muscles were highlighted with alpha smooth muscle actin. IHC. Bar = 100 μm.

Histologically the dermis was regionally expanded by a variably cellular, multilobulated proliferation of neoplastic cells that exhibited differentiation toward two primordial germ cell layers including ectoderm and mesoderm. For the ectoderm, there were areas of eosinophilic granular and fibrillar neuropil along with neural parenchyma of respective gray and white matter containing variable numbers of glial cells and neurons (Figure 2A), often intermingled with portions of mesenchymal tissue such as smooth muscle fibers or preexisting dermal collagen stroma. In some areas, there were variably sized well-circumscribed expansile follicular cysts that were lined by attenuated keratinizing squamous epithelium, and contained abundant keratin debris and numerous hair shafts. In the section through the teeth and associated alveolar bone, there was a large island of amphophilic dentin rimmed by ~50–70 μm thick, hyalinized, eosinophilic bands of enamel with an associated single palisading layer of tall columnar ameloblasts and variably loosely edematous periodontal ligament (Figure 2D). For the mesoderm, there were small, isolated lobules of well differentiated cartilage (Figure 3A), irregular trabeculae of bone (Figure 3B), often associated with marrow hematopoietic precursors (Figure 3C), bands of smooth muscle and myocardial fibers, and adipose tissue. High numbers of scattered melanocytes and melano-macrophages were seen throughout this region. Unfortunately, the proximal margin was composed entirely of a large portion of well differentiated lamellar vertebral bone, that contained large pools of hematopoietic precursors, and was surrounded by bundles of skeletal myofibers, nerve bundles, bulks of adipose tissue, and a lobule of myocardium in one focus. Thus, complete excision could not be definitively confirmed, histologically.

Tissues were immunostained for pan-cytokeratin (pan-CK, clone AE1/AE3, Dako, Carpinteria, CA, USA), vimentin (clone V9, Cell Marque, Rocklin, CA, USA), glial fibrillary acidic protein (GFAP, clone GA5, Sigma Aldrich, Saint Louis, MO, USA), neuron-n (clone 27-4, Millipore, Temecula, CA, USA), alpha smooth muscle actin (SMA, clone 1A4, Dako) and CD31 (clone H-3, Santa Cruz Biotechnology, Dallas, TX, USA). Squamous epithelial cells lining the epidermis, adnexal structures and follicular cyst showed strong cytoplasmic immunolabelling against pan-CK. In regions of neural tissue, several glial cells were immunoreactive for GFAP (Figure 2B). Some neurons are stained with neuron-n (Figure 2C). Smooth muscle fibers revealed strong cytoplasmic immunoreactivity for SMA (Figure 3D). Vimentin and CD31 highlighted lymphatic and blood vessels within the neoplasm. After 2 years post operation, no recurrent mass was detected at the surgical site.

## Discussion

Teratomas are complex neoplasms that derive from more than one embryonic germ layer. They can arise in either gonadal or extragonadal locations. It is widely accepted that extragonadal teratoma develops from primordial germ cells misplaced during their migration to gonads. The up- or down-regulation of *TFAP2C* and *PRDM1*, transcription factor genes, in misplaced germ cells could lead to altered epigenetics germline program and tumor formation (7). Teratomas have two variants including immature and mature forms. Mature teratomas are composed of well differentiated cells of two or three germ cell layers, while neoplasms with poorly differentiated embryonal components would be characterized as immature teratomas. Biological behavior can vary from benign to malignant, depending on the proportion between dedifferentiated or incompletely differentiated and well-differentiated cell populations. This unique tumor type has been reported in several animal species. In veterinary medicine, although gonadal teratomas are most common in bitches (8), rodents, particularly 129/Sv-ter+ mice (9), and guinea pigs (10), this tumor is rarely documented in other animal species such as horses (11) and heifers (12). Extragonadal teratomas are exceedingly rare, but have been reported in a variety of wildlife and domestic animals including ferrets (13), a juvenile Wistar rat (14), a dog (15) and domestic avian species such as a lesser kestrel (*Falco naumanni*) (16). In cats, gonadal teratomas including ovarian and testicular teratomas were unilateral and occurred at an age ranging from 5 months to 17 years (5, 6, 17, 18). Among these studies, mature teratomas had benign behavior and were curative after ovariohysterectomy (17, 18), while immature teratomas reported in two cats had malignant behavior with intra-abdominal metastasis and the ability to secrete estrogen (5, 6).

In humans, extragonadal teratomas were reported in the midline locations including sacrum, presacral space, coccyx, mediastinum, retroperitoneum, and intracranial space (19). The criteria indicating biological behavior and prognosis of extragonadal teratoma were age, sex, anatomic location, tumor size, immature histologic elements, coincident neoplasms, and cytogenetic abnormalities. Sacrococcygeal teratomas are most commonly occurred in childhood as congenital neoplasms. Mature sacrococcygeal teratomas showed a favorable outcome regardless of age and sex (20). In the previous cytogenetic study, sacrococcygeal teratomas had normal karyotype; however, high expression of gene was involved in early germ cell differentiation (*KIT, PRDM1*, and *TFAP2C*), and pluripotent factors (*ALPL* and *SOX2*). When compared between immature and mature of sacrococcygeal teratoma, immature group showed a different genetic expression pattern of ribosomes, histones, cytoskeleton and transcription regulators. On the other hand, mature sacrococcygeal teratoma had a higher gene activation of innate and adaptive immune effectors (21). In cats, most extragonadal teratomas were mature and benign and could be found anywhere in the body, such as intracranial (22), retrobulbar (1), temporal (2), perineal (3) of kitten or adult cats (age 4 months to 3 years old) except in the region of coccygeal vertebrae that had a mixture of mature and immature components (4). However, there was no recurrence observed after years of follow-up in all cases. While most teratomas can be diagnosed by gross and histopathologic examination, immunohistochemistry can be applied to confirm tissue differentiation and cells of origin, particularly in immature neoplasms (4).

In the present case, although endodermal elements such as respiratory and alimentary tissues were not observed. However, histologically, the presence of well-differentiated tissue of ectoderm and mesoderm within the neoplasm warrants a diagnosis of mature extragonadal teratoma at the base of the tail of a young cat. Additionally, several immunohistochemical markers including GFAP, neuron-n, pan-CK, vimentin and SMA aid the evaluation of degree of tissue differentiation. Comparing with the previous report of feline coccygeal teratoma, the present case also occurred at a similar age and location, with no evidence of invasion to the caudal vertebrae. In contrast, the previously reported case had all three primordial germ layers with immature tissue components highlighted by Sox-2. In this study, tail amputation should be a curative resection and there was no recurrent mass observed after 2 years of tail amputation. Taking all findings together, mature teratoma was confirmed and benign biological behavior was supposed. Although local recurrences or metastases are unlikely in this case, periodic monitoring of the surgical site remains recommended due to the unclean surgical margin.

## Data availability statement

The original contributions presented in the study are included in the article/supplementary material, further inquiries can be directed to the corresponding author.

## Ethics statement

Written informed consent was obtained from the owners for the participation of their animals in this study.

## Author contributions

SS and TK contributed to conception and design of the case description, wrote original draft, review, and editing. NS, NA, ST, and AR contributed to resources. All authors contributed to manuscript revision, read, and approved the submitted version.

## Funding

This study was partially supported by the Center of Excellence for Companion Animal Cancer, Department of Pathology, Faculty of Veterinary Science, Chulalongkorn University.

## Conflict of interest

The authors declare that the research was conducted in the absence of any commercial or financial relationships that could be construed as a potential conflict of interest.

## Publisher's note

All claims expressed in this article are solely those of the authors and do not necessarily represent those of their affiliated organizations, or those of the publisher, the editors and the reviewers. Any product that may be evaluated in this article, or claim that may be made by its manufacturer, is not guaranteed or endorsed by the publisher.
